# The prognostic value of radiomic features from pre- and post-treatment ^18^F-FDG PET imaging in patients with nasopharyngeal carcinoma

**DOI:** 10.1038/s41598-023-35582-x

**Published:** 2023-05-25

**Authors:** Soo Jeong Kim, Joon Young Choi, Yong Chan Ahn, Myung-Ju Ahn, Seung Hwan Moon

**Affiliations:** 1 Department of Nuclear Medicine, Kangbuk Samsung Hospital, Sungkyunkwan University School of Medicine, 29, Saemunan-ro, Jongno-gu, Seoul, 03181 Republic of Korea; 2 Department of Nuclear Medicine, Samsung Medical Center, Sungkyunkwan University School of Medicine, 81, Irwon-ro, Gangnam-gu, Seoul, 06351 Republic of Korea; 3 Department of Radiation Oncology, Samsung Medical Center, Sungkyunkwan University School of Medicine, Seoul, Republic of Korea; 4 Division of Hematology-Oncology, Department of Medicine, Samsung Medical Center, Sungkyunkwan University School of Medicine, Seoul, Republic of Korea

**Keywords:** Cancer, Biomarkers, Oncology

## Abstract

Positron emission tomography/computed tomography (PET/CT) with ^18^F-fluorodeoxyglucose (FDG) is widely used for management of nasopharyngeal carcinoma (NPC). Combining the radiomic features of pre- and post-treatment FDG PET images may improve tumor characterization and prognostic predication. We investigated prognostic value of radiomic features from pre- and post-radiotherapy FDG PET images in patients with NPC. Quantitative radiomic features of primary tumors were extracted from the FDG PET images of 145 NPC patients and the delta values were also calculated. The study population was divided randomly into two groups, the training and test sets (7:3). A random survival forest (RSF) model was adopted to perform analyses of progression-free survival (PFS) and overall survival (OS). There were 37 (25.5%) cases of recurrence and 16 (11.0%) cases of death during a median follow-up period of 54.5 months. Both RSF models with clinical variables and radiomic PET features for PFS and OS showed comparable predictive performance to RSF models with clinical variables and conventional PET parameters. Tumoral radiomic features of pre- and post-treatment FDG PET and the corresponding delta values may predict PFS and OS in patients with NPC.

## Introduction

Nasopharyngeal carcinoma (NPC) is an uncommon epithelial carcinoma and has characteristics distinct from those of other epithelial head and neck cancers. Globally, NPC shows an unbalanced geographic distribution and the non-keratinizing subtype of NPC, which is associated with Epstein-Barr virus (EBV) infection, is predominant in EBV-endemic areas^1^. Radiotherapy is mainstay of NPC treatment due to its radio-sensitive nature and anatomic location of the tumors^2,3^. Improvement of clinical outcomes among patients with NPC has been attributed to advances in radiotherapy techniques^4^. Meanwhile, the effects of radiotherapy treatment may persist for months and are accompanied by inflammation^5^, which makes it difficult to evaluate early treatment response.

Positron emission tomography/computed tomography (PET/CT) with ^18^F-fluorodeoxyglucose (FDG) is a widely used imaging modality for management of NPC. In addition, FDG PET/CT is used to predict the prognosis of patients with NPC. High maximum standardized uptake value (SUV), metabolic tumor volume (MTV) and total lesion glycolysis (TLG), which are major conventional parameters derived from SUV, in pre-treatment FDG PET/CT images of patients with NPC have been associated with adverse prognosis in a meta-analysis^6^. Also, significant residual FDG uptake in post-radiotherapy FDG PET/CT images has been associated with poor clinical outcomes^7^. However, the predictive value of various FDG PET parameters, which could provide abundant information about tumor biology, remain to be clarified with regard to treatment response and prognosis.

Intratumoral heterogeneity (ITH) implies subclonal cell variability in time and space, and involves genetic, epigenetic, and metabolic features^8^. A high degree of ITH is known to be associated with inferior clinical outcomes and may contribute to the development of resistance and treatment failure in cancer patients^9^. Various FDG PET texture parameters could be used to evaluate metabolic ITH non-invasively^10^. Furthermore, difference in the features obtained from pre- and post-treatment images could reveal subtle changes in the tumor^11^. Combining the radiomic features of pre- and post-treatment FDG PET images may improve tumor characterization and prognostic prediction in patients with NPC. However, there has been no previous study using FDG PET to evaluate the radiomic features of NPC tumors both pre- and post-treatment.

In this study, we assessed the prognostic value of radiomic features identified in pre- and post-radiotherapy FDG PET images among patients with NPC.

## Methods

### Study subjects

**Figure 1:**
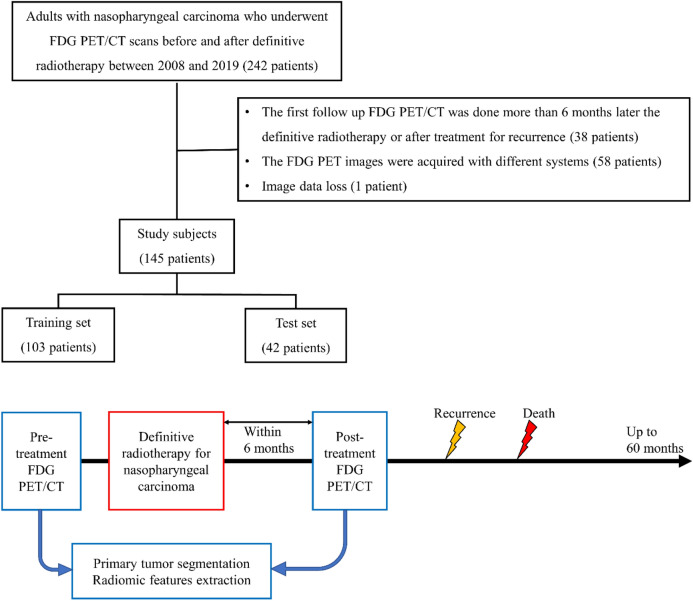
Flow chart of study subjects and FDG PET processing.

All patients included in this study were diagnosed with NPC and underwent FDG PET/CT scans before and after definitive radiotherapy (with or without chemotherapy), between 2008 and 2019. The study population also met the following criteria: (1) subjects were more than 20 years of age; (2) the interval between definitive radiotherapy and follow-up FDG PET/CT was less than 6 months; and (3) the PET images were acquired with the same spatial resolution within the study population. We excluded patients who were treated for recurrence before the follow-up FDG PET/CT. Patients with the PET/CT image data loss were also excluded. Consequently, 145 patients were included in this study. Figure 1 is flow diagram of study subjects and FDG PET processing.

This study was conducted in accordance with the Declaration of Helsinki and approved by the Institutional Review Board of Samsung Medical Center (protocol code 2020-06-127; date of approval: 15 July 2020). The requirement of written informed consent from enrolled subjects was waived by the Institutional Review Board of Samsung Medical Center due to the retrospective study design.

### Medical record review

In this retrospective study, medical records were reviewed for demographic/clinical characteristics and information on recurrence/death among NPC patients. Follow-up of records review included the 60 months after definitive radiotherapy. Progression-free-survival (PFS) was defined as the duration of definitive radiotherapy completion and recurrence in months. Date of recurrence was determined as the date of documented clinical decision based on imaging modalities. Overall survival (OS) was defined as the duration to definitive radiotherapy completion and death from all causes in months. Cancer staging was based on the Sixth Edition of the American Joint Committee on Cancer’s Cancer Staging Manual to 2009, the Seventh Edition from 2010 to 2017, and Eighth Edition after 2018.

### FDG PET/CT imaging

Patients fasted for at least 6 h before the injection of FDG, and their blood glucose concentration was confirmed to be less than 200 mg/dL at the time of FDG injection. At 60 min after injection of 5 MBq/kg of FDG, imaging was performed using an STe PET/CT scanner (GE Healthcare) without intravenous or oral contrast. Whole-body CT images were obtained with a continuous spiral 16-slice helical CT technique (140 keV; 30–170 mA; section width, 3.75 mm). An emission PET scan was obtained from the level of the thigh to the skull base. Scanning was performed at 2.5 min per frame in 3-D mode with attenuation-corrected images (3.9 × 3.9 × 3.3 mm) reconstructed using a 3-D ordered-subset expectation maximization algorithm (20 subsets, 2 iterations).

### Imaging analyses

Primary tumor segmentation of all pre- and post-treatment FDG PET images was computed using the gradient-based segmentation method (‘PET edge’) of MIM version 6.4 (MIM software, Inc., Cleveland, OH, USA). Only primary tumors were analyzed in this study. A total of 72 quantitative radiomic features were extracted from the PET images using Chang-Gung Image Texture Analysis (CGITA) toolbox^12^. The relative variation of each radiomic feature was also evaluated in pre- and post-treatment scans:

$$\Delta\,{\text{feature}} = \frac{{feature_{post - treatment} - feature_{pre - treatment} }}{{feature_{pre - treatment} }}$$

### Statistical analyses

The study population was divided randomly into two groups, the training and test sets (7:3) using the createDataPartition function of caret package in R. The demographic and clinical characteristics were compared between two groups using Fisher’s exact test, the chi-square test, and Wilcoxon rank sum test. Two-sided p values less than 0.05 were considered significant. A random survival forest (RSF) model was adopted to perform survival analyses of PFS and OS in the training set. An RSF is a nonparametric method to analyze right-censored survival data with multiple covariates and there is no need to fulfill any assumptions such as the proportional hazards assumption of classical Cox regression^13^. An RSF provides a variable importance (VIMP) that demonstrates variables of key role in predicting the survival outcome. First, we developed an RSF model with clinical variables—age, sex, smoking history, p16 status, EBV status, and stage— and conventional PET parameter—maximum SUV, mean SUV, MTV, and TLG—from pre- and post-treatment scans. Next, we made another RSF model with clinical variables and selected 10 relatively important radiomic PET features. Each model was then evaluated in the test set. Brier score (BS; mean squared error) was used to assess the prediction performance of the models in the training and test sets. Time-dependent BS and continuous ranked probability scores (CRPS; integrated BS divided by time) were recorded.

All statistical analyses were performed using R version 4.1.2 (The R Foundation for Statistical Computing, Vienna, Austria), with caret version 6.0.92, dplyr version 1.0.9, psych version 2.2.5, tidyverse version 1.3.1, gmodels version 2.18.1.1., survival version 3.3.1, randomForestSRC version 3.1.0 packages.

## Results

### Study subjects’ characteristics

The characteristics and clinical outcomes of 145 study subjects are summarized in Table 1. The median age was 52.3 (range 20.2–80.7) years and 108 of 145 (74.5%) subjects were males. There were 37 (25.5%) cases of recurrence and 16 (11.0%) deaths, respectively, during a median follow-up period of 54.5 months. Demographic and clinical characteristics of the training and test groups were not significantly different.

**Table 1:** Characteristics of subjects.

	All (n = 145)	Training set (n = 103)	Test set (n = 42)	*p* value
Age				
Median (range)	52.3 (20.2–80.7)	51.8 (20.2–80.7)	53.3 (34.2–76.1)	0.331^a^
Sex				
Male	108 (74.5%)	75 (72.8%)	33 (78.6%)	0.471^b^
Female	37 (25.5%)	28 (27.2%)	9 (21.4%)	
Smoking history				
Yes	81 (55.9%)	57 (55.3%)	24 (57.1%)	0.842^b^
No	45 (31.0%)	32 (31.1%)	13 (31.0%)	0.989^b^
Unspecified	19 (13.1%)	14 (13.6%)	5 (11.9%)	0.785^b^
EBV				
Positive	38 (26.2%)	30 (29.1%)	8 (19.0%)	0.211^b^
Negative	1 (0.7%)	0	1 (2.4%)	0.290^c^
Unspecified	106 (73.1%)	73 (70.9%)	33 (78.6%)	0.343^b^
p16 status				
Positive	2 (1.4%)	1 (1.0%)	1 (2.4%)	0.497^c^
Negative	6 (4.1%)	4 (3.9%)	2 (4.8%)	1.000^c^
Unspecified	137 (94.5%)	98 (95.1%)	39 (92.9%)	0.691^c^
T stage				
T1	57 (39.3%)	45 (43.7%)	12 (28.6%)	0.258^b^
T2	22 (15.2%)	16 (15.5%)	6 (14.3%)	
T3	42 (29.0%)	28 (27.2%)	14 (33.3%)	
T4	24 (16.6%)	14 (13.6%)	10 (23.8%)	
N stage				
N0	23 (15.9%)	19 (18.4%)	4 (9.5%)	0.269^b^
N1	51 (35.2%)	37 (35.9%)	14 (33.3%)	
N2	53 (36.6%)	33 (32.0%)	20 (47.6%)	
N3	18 (12.4%)	14 (13.6%)	4 (9.5%)	
M stage				
M0	143 (98.6%)	102 (99.0%)	41 (97.6%)	0.405^c^
M1	2 (1.4%)	1 (1.0%)	1 (2.4%)	
Overall stage				
I	10 (6.9%)	8 (8.8%)	2 (4.8%)	0.659^c^
II	36 (24.8%)	28 (27.2%)	8 (19.0%)	
III	57 (39.3%)	39 (37.9%)	18 (42.9%)	
IV	42 (29.0%)	28 (27.2%)	14 (33.3%)	
Radiotherapy				
Cumulative dose (cGy), median (range)	6840 (6120–7240)	6840 (6600–7240)	6840 (6120–7000)	0.932^a^
Fraction, median (range)	30 (27–35)	30 (28–35)	30 (27–35)	0.440^a^
Chemotherapy				
Induction	1 (0.7%)	0	1 (2.4%)	0.290^c^
Concurrent	132 (91.0%)	92 (89.3%)	40 (95.2%)	0.348^c^
Adjuvant	11 (7.6%)	9 (8.7%)	2 (4.8%)	0.511^c^
Recurrence				
Yes	37 (25.5%)	26 (25.2%)	11 (26.2%)	0.905^b^
Censored	108 (74.5%)	77 (74.8%)	31 (73.8%)	
PFS				
Months, median (range)	48.6 (2.4–60)	48.1 (2.4–60)	50.8 (3.9–60)	0.654^a^
Death				
Yes	16 (11.0%)	9 (8.7%)	7 (16.7%)	0.240^c^
Censored	129 (89.0%)	94 (91.3%)	35 (83.3%)	
OS				
Months, median (range)	54.6 (4.3–60)	54.4 (4.3–60)	55.6 (20.3–60)	0.863^a^

^a^Wilcoxon rank sum test.

^b^Chi-square test.

^c^Fisher’s exact test.

### Analyses of PFS

The RSF analyses with clinical variables and conventional PET parameters for PFS using the training set resulted in an out-of-bag (OOB) error rate of 49.2%. On the other hand, Δcode similarity (texture feature coding co-occurrence), Δinverse difference moment (texture feature coding co-occurrence), mean SUV_post-treatment_, code similarity (texture feature coding co-occurrence)_pre-treatment_, high-intensity zone emphasis (intensity-size-zone)_post-treatment_, high-intensity short-zone emphasis (intensity-size-zone)_post-treatment_, Δhomogeneity (texture feature coding co-occurrence), entropy (normalized co-occurrence)_pre-treatment_, inverse difference moment (texture feature coding co-occurrence)_post-treatment_, and high-intensity run emphasis (voxel-alignment)_post-treatment_ were relatively important in the RSF analyses with all radiomic PET parameters. The VIMP of the radiomic PET parameters is summarized in Table 2. After that, an RSF model with clinical variables and ten selected radiomic PET parameters was developed using the training set, and the OOB error rate was 30.4%.

**Table 2:** Importance of each radiomic PET variable in predicting PFS.

	Variable importance	Relative importance
Δcode similarity (texture feature coding co-occurrence)	0.0340	1.0000
Δinverse difference moment (texture feature coding co-occurrence)	0.0293	0.8634
Mean SUV_post-treatment_	0.0247	0.7283
Code similarity (texture feature coding co-occurrence)_pre-treatment_	0.0177	0.5201
High-intensity zone emphasis (intensity-size-zone)_post-treatment_	0.0155	0.4568
High-intensity short-zone emphasis (intensity-size-zone)_post-treatment_	0.0123	0.3624
Δhomogeneity (texture feature coding co-occurrence)	0.0113	0.3328
Entropy (normalized co-occurrence)_pre-treatment_	0.0107	0.3149
Inverse difference moment (texture feature coding co-occurrence)_post-treatment_	0.0093	0.2730
High-intensity run emphasis (voxel-alignment)_post-treatment_	0.0092	0.2703

**Figure 2:**
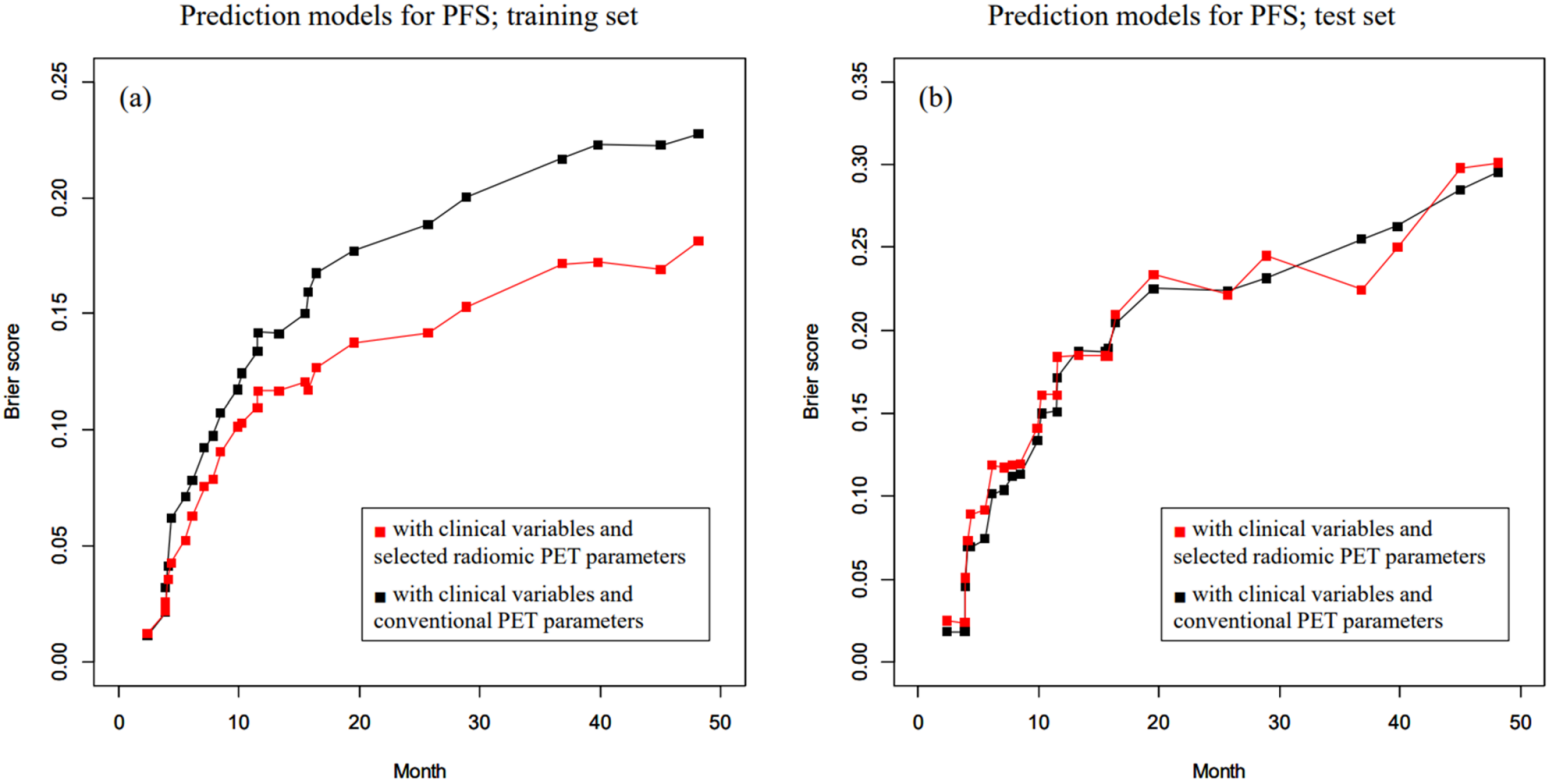
Time-dependent Brier score (BS; mean-squared error) curve of random survival forest models to predict of progression-free survival (PFS). (**a**) The predictive performance of the model with clinical variables and selected radiomic PET parameters was superior to that of the predictive model with clinical variables and conventional PET parameters when using the training set. (**b**) The predictive performance of model with clinical variables and selected radiomic PET parameters was comparable to the model with clinical variables and conventional PET parameters when using the test set.

The CRPS of the predictive model for PFS with clinical variables and conventional PET parameters was 0.165 when using the training set and 0.199 when using the test set. In addition, the CRPS of the predictive model for PFS with clinical variables and selected radiomic PET parameters was 0.128 when using the training set and 0.200 when using the test set. The time-dependent BS for each prediction model is provided in Fig. 2.

### Analyses for OS

**Figure 3:**
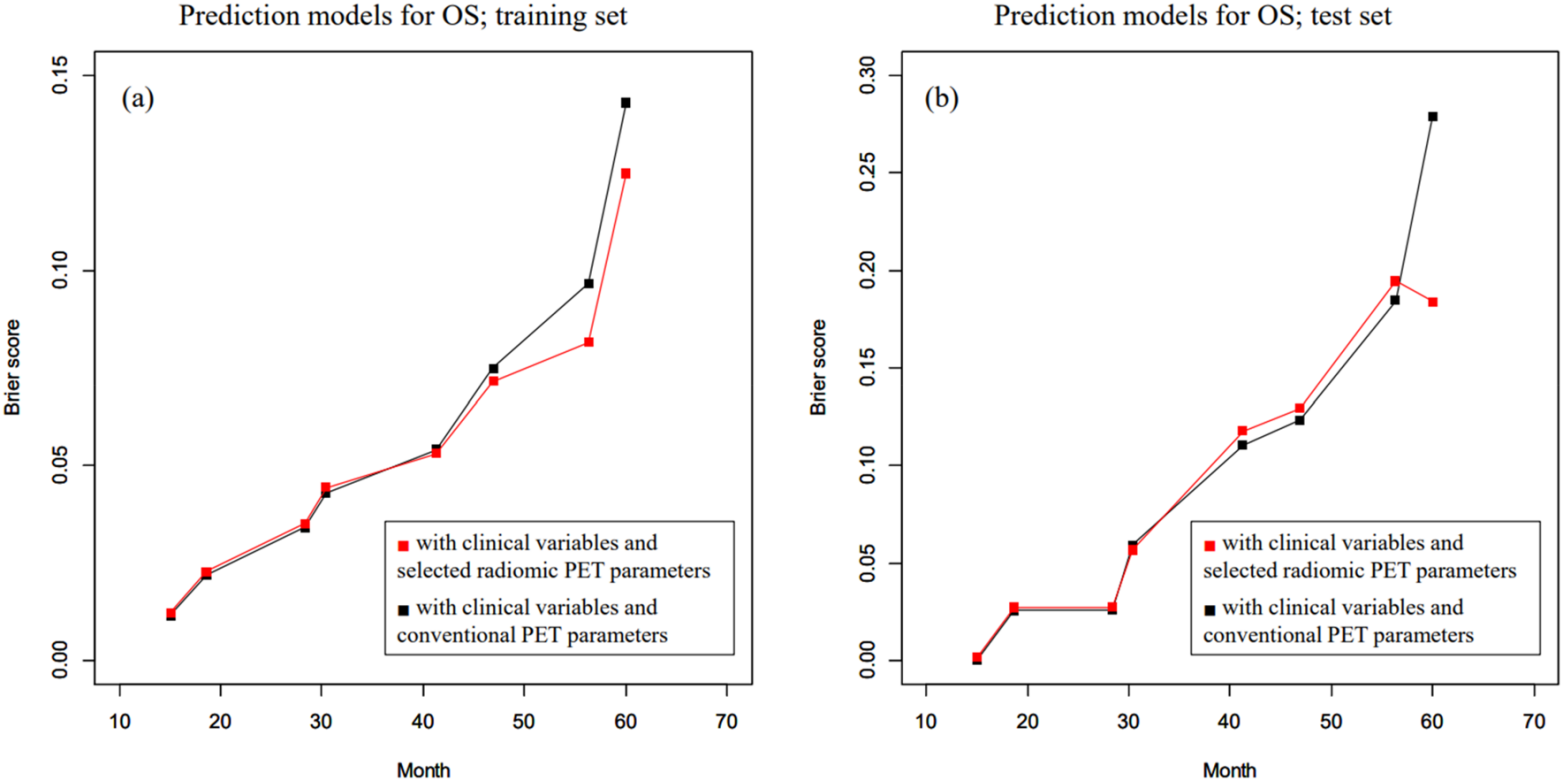
Time-dependent Brier score (BS; mean-squared error) curve of random survival forest models to predict overall survival (OS). (**a**) The predictive performance of the model with clinical variables and selected radiomic PET parameters was better than that of the predictive model with clinical variables and conventional PET parameters when using the training set. (**b**) The predictive performance of the model with clinical variables and selected radiomic PET parameters was similar to that of the model with clinical variables and conventional PET parameters when using the test set.

The RSF analyses with clinical variables and conventional PET parameters for OS when using the training set resulted in an OOB error rate of 35.2%. On the other hand, coarseness (neighborhood intensity-difference)_pre-treatment_, tumor volume (SUV statistics)_pre-treatment_, inverse difference moment (texture feature coding co-occurrence)_post-treatment_, run-length variability (voxel-alignment)_pre-treatment_, code similarity (texture feature coding co-occurrence)_post-treatment_, zone percentage (intensity-size-zone)_post-treatment_, minimum SUV (SUV statistics)_pre-treatment_, inverse difference moment (co-occurrence)_pre-treatment_, Δlow-intensity zone emphasis (intensity-size-zone), and max spectrum (texture spectrum)_pre-treatment_ were relatively important in RSF analysis with all radiomic PET parameters for OS. The VIMP of the radiomic PET parameters is summarized in Table 3. An RSF model with clinical variables and the selected radiomic PET parameters for OS was developed using the training set and the OOB error rate was 31.6%. The CRPS of the prediction model for OS with clinical variables and conventional PET parameters was 0.043 when using the training set and 0.071 when using the test set. In addition, the CRPS of the predictive model for OS with clinical variables and selected radiomic PET parameters was 0.040 when using the training set and 0.071 when using the test set. The time-dependent BS for each predictive model is demonstrated in Fig. 3.

**Table 3:** Importance of each radiomic PET variable in predicting OS.

	Variable importance	Relative importance
Coarseness (neighborhood intensity-difference)_pre-treatment_	0.0510	1.0000
Tumor volume (SUV statistics)_pre-treatment_	0.0327	0.6408
Inverse difference moment (texture feature coding co-occurrence)_post-treatment_	0.0178	0.3483
Run-length variability (voxel-alignment)_pre-treatment_	0.0130	0.2549
Code similarity (texture feature coding co-occurrence)_post-treatment_	0.0121	0.2373
Zone percentage (intensity-size-zone)_post-treatment_	0.0099	0.1944
Minimum SUV (SUV statistics)_pre-treatment_	0.0086	0.1692
Inverse difference moment (co-occurrence)_pre-treatment_	0.0078	0.1521
Δlow-intensity zone emphasis (intensity-size-zone)	0.0074	0.1459
Max spectrum (texture spectrum)_pre-treatment_	0.0067	0.1320

## Discussion

To the best of our knowledge, this is the first study to evaluate the prognostic value of tumoral radiomic features from pre- and post-radiotherapy FDG PET images in patients with NPC. Texture parameters from pre- and post-treatment FDG PET scans had an impact on prediction of the prognosis of NPC patients. RSF models with clinical variables and radiomic PET features showed comparable prediction performance for PFS and OS to RSF models with clinical variables and conventional PET parameters.

The prognostic value of textural features derived from pre-treatment FDG PET/CT imaging of head and neck squamous cell carcinoma (HNSCC) has been evaluated in several previous studies. Yoon et al. conducted univariate and multivariate regression analysis of 119 cases of HNSCC and reported that pre-treatment FDG PET radiomics contain prognostic information^14^; however, the tumors were mainly located in the oropharynx (71.4%) in that study, while only 5 NPC patients were included. Similarly, Feliciani G. et al. analyzed 90 HNSCC patients and reported the superiority of imaging biomarkers from a pre-treatment FDG PET/CT model over a clinical-variables-alone model for prediction of PFS^15^; nevertheless, only 13 (15%) NPC patients were included in the study. Given the distinctive character of NPC, we included only NPC patients in this study rather than all HNSCC patients. In addition, Peng et al. developed models to predict treatment failure (locoregional recurrence or distant metastases) in 85 locally advanced NPC patients using machine learning methods^16^. They reported that a model with only PET parameters demonstrated greater area under curve (AUC) values for predictive performance than models with only clinical variables or with PET and clinical variables. The Peng et al. considered whether adverse events occurred or not, and the follow-up duration was not standardized in the retrospective study. We analyzed PFS and OS to avoid possible selection bias due to loss to follow-up.

Among radiomic PET features, Δcode similarity (texture feature coding co-occurrence) was an important variable for PFS prediction and coarseness (neighborhood intensity-difference)_pre-treatment_ was an important variable for OS prediction in this study. Code similarity is based on texture feature number (TFN) co-occurrence matrix and demonstrates the density of same TFNs in its 8 connectivity neighborhood^17^. Lower pretreatment tumor code similarity was reported to be associated with poor survival in patients with esophageal squamous cell carcinoma^18,19^. On the other hand, coarseness (neighborhood-intensity difference) is based on the differences between each voxel and the neighboring voxels in the adjacent image planes and associated with the human perception of image granularity^20^. Greater coarseness_neighbouring gray tone difference matrix (NGTDM) was reported to be associated with higher risk of treatment failure in locally advanced NPC patients^16^. Coarseness was also negatively correlated with OS in a cohort study with various HNSCC patients^21^.

Delta radiomic features on serial images are longitudinal changes in radiomic features and were introduced to assess treatment response noninvasively when using various imaging modalities to assess various cancers^11,22–24^. A previous study investigated the potential of delta radiomics when using quantitative ultrasound to assess early treatment response during radiotherapy in HNSCC based on the index lymph node^25^. Response prediction accuracy was 86% using delta radiomics features acquired one week into radiotherapy, though the study had a limitation, in that primary tumors could not be accessed by ultrasound. In this study, we assessed primary tumors that were located at depth using FDG PET/CT, and found delta-radiomic features were important variables, especially for predicting PFS. Delta radiomics with FDG PET/CT may improve prediction of the prognosis of patients with NPC.

Our study has several limitations. First, the study was conducted in a relatively small number of subjects at a single center. Further study with more subjects in multiple centers is needed to develop a more precise predictive model of the survival of NPC patients and generalize the model. We intended to assess the potential prognostic power of tumor radiomic features in this preliminary study. In addition, the follow-up period after definitive treatment in this retrospective study was restricted to 60 months, because most patients who had survived for 5 years without recurrence had no further clinical visits to our institution. Missing data in smoking history, EBV, and p16 status arise from retrospective study design may obscure clinical implication of the variables. Furthermore, pre-treatment plasma EBV deoxyribonucleic acid (DNA) is an established prognostic factor for EBV-related NPC^26^. However, data regarding plasma EBV DNA titers was not available in this study. We did not consider the differences in detailed treatment strategies between subjects, either. We thus conducted analyses with clinical variables and conventional PET parameters in addition to the analyses of interest, which comprised clinical variables and selected radiomic PET parameters, to serve as a comparison.

## Conclusions

Tumoral radiomic features of pre- and post-treatment FDG PET images and the corresponding delta values may contain prognostic values for PFS and OS in patients with NPC.

## Data Availability

The datasets used and/or analyzed during the current study available from the corresponding author on reasonable request.
